# Assessment of anterior positioning splint in conjunction with lateral pterygoid BTX injection to treat TMJ disc displacement with reduction — a preliminary report

**DOI:** 10.1186/s40902-021-00317-3

**Published:** 2021-09-08

**Authors:** Maram Taema, Nouran Abdel Nabi, Samira Ibrahim, Heba Ahmed Kamal, Aala’a Emara

**Affiliations:** 1 Faculty of Dentistry, Department of Prosthoontics, Cairo University, Cairo, Egypt; 2 Faculty of Medicine, Department of Diagnostic and Interventional Radiology, Cairo University, Cairo, Egypt; 3 Faculty of Dentistry, Oral & Maxillofacial Surgery Department, Cairo University, Cairo, Egypt

**Keywords:** TMJ, BTX, Disc displacement with reduction, Anterior positioning splint, Lateral pterygoid muscle

## Abstract

**Objective:**

Treatment of temporomandibular disc displacement with reduction is controversial. This study assesses the use of an anterior positioning splint with botulinum toxin in the lateral pterygoid muscle (BTX) for such cases.

**Methods:**

Twelve joints were included; groups I and II received BTX injection while group II also received an anterior positioning splint. Pain scores and clicking status were recorded at regular intervals then a postoperative MRI was done after 4 months.

**Results:**

Clinical improvement was noted in both groups. Mean pain scores dropped significantly and clicks in the twelve joints disappeared in 83% of group I and 33% of group II. MRIs showed significant disc position improvement with the higher mean change (1.33 ± 0.76) in group I.

Group I showed better improvement of discal position and only one joint regained a click. Patients of group II reported discomfort from the splint which may have caused psychological distress and so worst pain scores.

**Conclusions:**

Group I  showed slightly better results but the cost of BTX injections and the complications of the splint should be kept in mind and the decision of treatment selection made according to each condition.

## Introduction

Temporomandibular disorders (TMDs) are considered the second most common musculoskeletal condition causing pain and functional disability [1]. The pain associated with TMDs is of a mild and fluctuating character that is aggravated by chewing or other jaw functions. The aetiology of these disorders is still controversial and different theories have been proposed; mechanical displacement, neuromuscular, psychophysiological, muscular, and psychological theories [2]. Mechanical interferences may be caused by a deformed or displaced articular disc. Internal derangements of the disc are caused by deviations in the anatomical position or form of tissues within the joint capsule which are only clinically noted when they cause movement interferences such as a click or a locking joint. Internal derangements include anterior disc displacement (ADD) where the disc is anteriorly displaced from its normal position to settle between the condyle and the articular eminence, but regains its position over the condylar head during mandibular opening. Although some cases do not require treatment; the sudden onset of clicking sounds from the joint accompanied by jaw instability cause patient anxiety [3]. Pharmacological and splint therapy have been reported to manage such cases with mixed reports of success [4]. This led to the introduction of combination management protocols to treat such cases.

Botulinum toxin (BTX) plays a role in the management of a variety of medical conditions, including dystonia, haemifacial spasm, various spastic movement disorders, headaches, hypersalivation, and hyperhidrosis. Botulinum toxin in maxillofacial muscles for treatment of pain, tonicity changes, and improving the symptoms of TMDs has been discussed in literature [5]. Von Lindren’s group evaluated the effect of BTX injections on reducing the muscle pain associated with temporomandibular joint dysfunction. Electromyography (EMG) was used during injection in muscles that were difficult to access including the lateral pterygoid muscle which was approached extra-orally in this study. The group reported that pain improved in 80% of the patients, while by the end of the observation period, 17% of patients had to receive a second injection because of recurrent pain [6]. BTX was recommended for patients who did not respond to conservative treatment options such as pharmacologic and physical methods [7]. Bakke et al. introduced a novel technique where BTX was injected intra orally into the lateral pterygoid muscle in cases suffering from ADD with reduction. The hypothesis was that the lateral pterygoid muscle’s hyperactivity was responsible for the anterior pull on the disc causing its displacement. In the follow-up period, they reported complete resolution of the click [5]. Later, Emara et al. used the same technique on a larger sample size and concluded that BTX injection in the lateral pterygoid muscle also led to the disappearance of the joint clicking clinically and a significant improvement in the disc position when viewed on the magnetic resonance imaging (MRI) [8]. This supported the theory proposing the lateral pterygoid muscle’s responsibility for anterior TMJ disc displacement. A published literature review concluded that BTX injection in the lateral pterygoid muscle reduced joint clicking and other TMD symptoms but added that blinded clinical trials assessing the effect of the BTX injection in the lateral pterygoid muscle are necessary [9]*.* This study therefore aims to evaluate the efficacy of botulinum toxin type A injection into the lateral pterygoid muscle, with and without an anterior positioning splint in patients with anterior disc displacement with reduction.

## Materials and methods

### Patient selection

Six fully dentate, motivated patients diagnosed with ADD with reduction suffering from painful clicking joints were selected with no other concomitant masticatory disorders/myogenic disorders. A total number of twelve joints were included in the study with an age range of 18–35. The patients went through meticulous preoperative examination (history, dental examination, medical history, clinical examination including muscle, and joint assessments) to reach the definitive diagnosis. Exclusion criteria include pregnant females, patients with pacemakers, masticatory parafunctional habits, or arthritic/osteophytic signs. They were informed of the study steps, possible complications, necessary commitment, and consented to be enrolled. The patients were then randomly divided into two groups using RANDOM.ORG. An ethical approval was acquired from the Research Ethics Committee at the Faculty of Dentistry, Cairo University (approval number REC_30.12.2014), and the RCT complied with the Declaration of Helsinki.

### Clinical steps

Patients in both groups of the study received identical BTX injection in the lateral pterygoid muscle, while group II also got an anterior positioning splint one week after BTX injection. After detailed history taking, preoperative measurements of the mandibular range of movement (maximal interincisal opening and lateral excursion) were recorded using the ORAstretch (OraStretch trademark of CranioMandibular Rehab, Inc., USA). The maximal interincisal opening here was the maximal unassisted opening. The NRS (numerical rating scale) was used to record the level of pain with 10 the highest and 1 the lowest grades of pain.

The selected patients were scheduled for MRI examination in closed and maximal opening positions in the sagittal-oblique plane on a multiplanar machine (Philips Achieva MRI 1.5 T Koninkliile Philips, Netherlands). The images were saved in digital form on a personal computer for analysis. The most centralized proton density (PD) images were selected for further editing and processing.

#### Botox injection

**Fig. 1:**
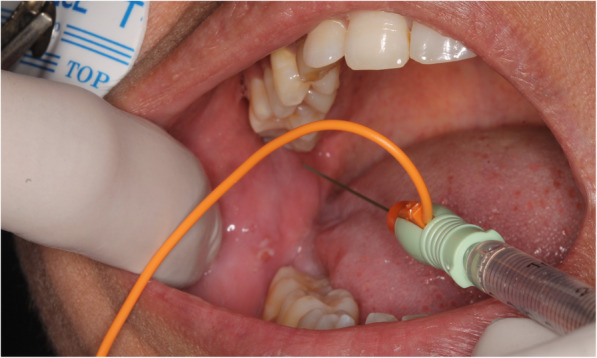
Photograph showing the electromyograph-guided injection of botulinum toxin solution into the lateral pterygoid muscle via an intraoral approach

The BTX vial [100 IU ALLERGAN (Allergan, USA) — Botox type A] was unpacked and 2 ml saline added slowly along the walls of the vial to obtain a 5 unit/0.1 ml solution. 0.7 ml of the solution — containing 35 U BTX — was drawn into an insulin syringe and an audible electromyogram (EMG)^1^ was used during injection to confirm needle position at the insertion of the lower head of the lateral pterygoid muscle. The lateral pterygoid muscle was approached intraorally from the opposite side and advanced lateral to the maxillary tuberosity with the needle directed towards the neck of the condyle, where it inserts into the neck of the condyle. The EMG produced a distinct loud sound confirming position within muscular tissue on function. After negative aspiration, the solution was injected slowly over a period of 5–10 s (Fig. 1).

#### Splint fabrication

**Fig. 2:**
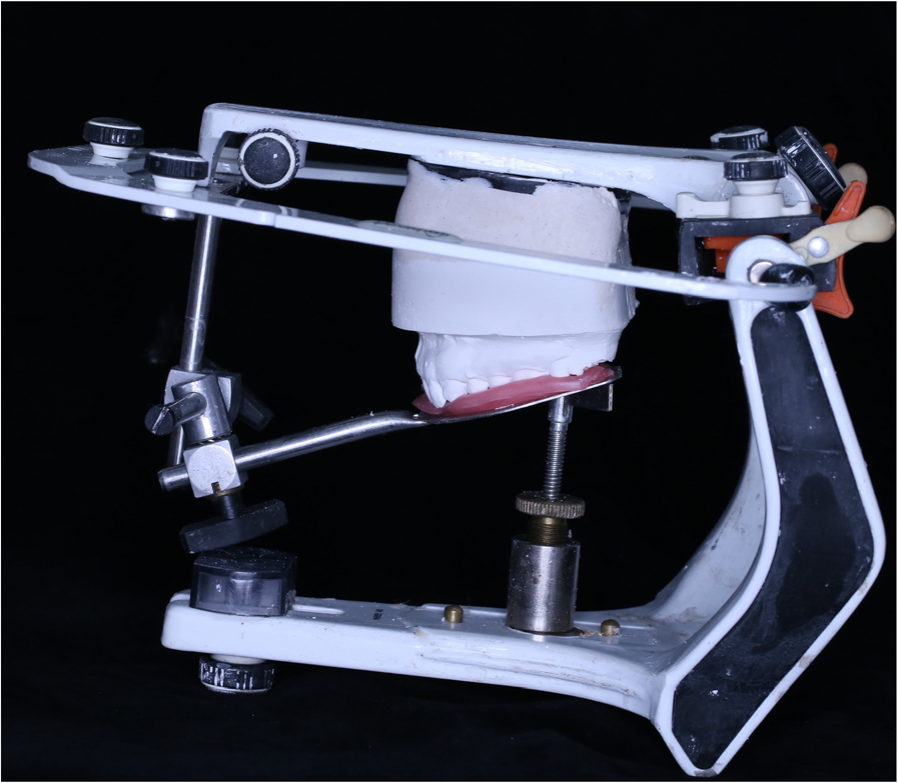
Transfer of the face-bow record to the semi-adjustable articulator to allow for accurate mandibular cast mounting

**Fig. 3:**
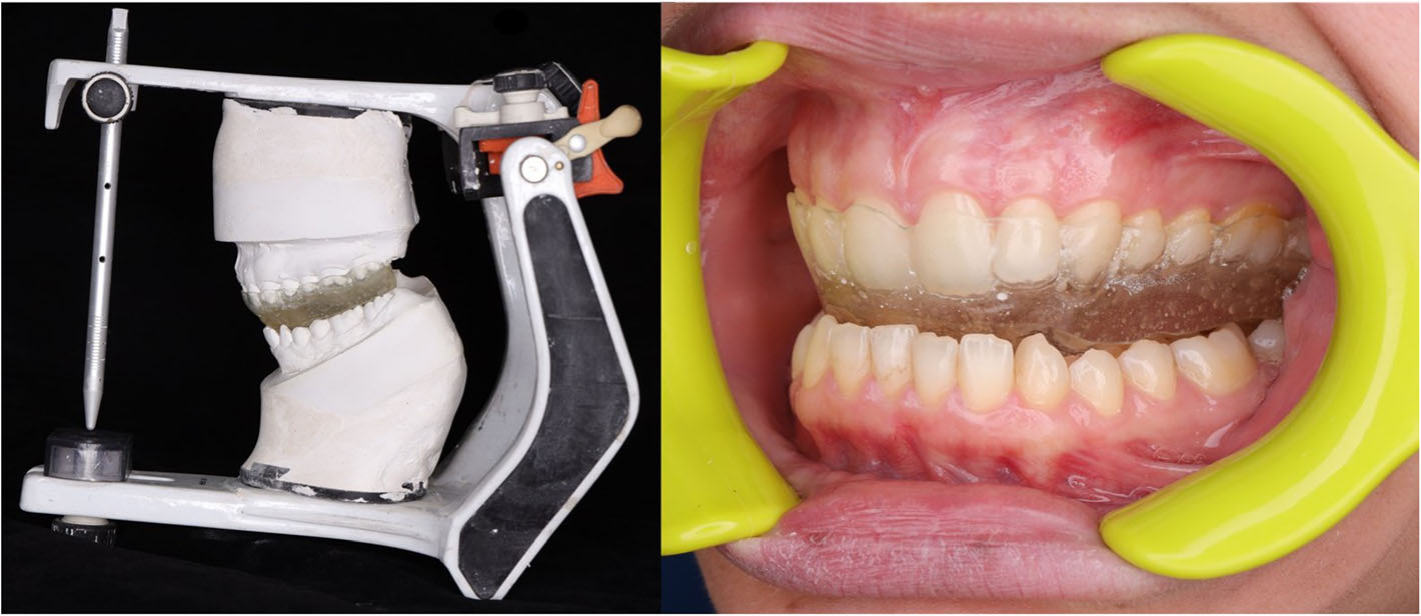
Left: Fabrication of the anterior positioning splint on a semi-adjustable articulator to achieve an anterior mandibular position which eliminates the click. Right: Intraoral photograph of finished splint in position and the anterior mandibular position

Maxillary and mandibular primary impressions were made for all the patients in group II using alginate impression material in stock trays and poured with type III hard dental stone. The maxillary cast was duplicated with alginate mix and the original cast was used to form a vacuum-formed acrylic splint (2-mm-thick acrylic resin). The duplicated maxillary cast was then mounted using face-bow transfer on a semi-adjustable articulator (Bio-Art Equipmentos Odontologicos Ltda., Brazil) (Fig. 2). The mandibular cast was mounted — according to the bite registration record — in the downward and anterior position at which the clicking disappeared. The vacuum-formed splint was seated on the maxillary cast and its occlusal surfaces roughened using a diamond stone. Separating medium was applied on the occlusal surface of the lower teeth and transparent self-cure acrylic resin applied to the occlusal surface of the maxillary splint anteriorly and posteriorly. The articulator was closed and after setting of the resin material the maxillary splint was removed with the acrylic ramp created anteriorly. Areas of tooth contact on the ramp were marked and the rest of the guiding planes removed; except the anterior portion to guide the patient’s mandible to the targeted anterior position. The splint was checked intraorally for proper adaptation, patient comfort, and balanced occlusion, and it was then smoothened and polished (Fig. 3). Instructions and importance of proper adherence to splint wearing were conveyed to the patients. The patients were instructed to wear their splint only during sleep and slight discomfort for the first few days was to be anticipated. Daily cleansing of the splint and maintenance of proper oral hygiene measures were also recommended (Fig. 2).

### Post-injection assessment

The patients of both groups were recalled for follow-up 2 weeks, 1 month, and 2, 3, and 4 months after the BTX injection. The same preoperative measurements were recorded in each of the follow-up sessions. MR assessment of identical parameters was ordered 4 months later and saved in digital format.

The pre-injection and post-injection magnetic resonance images were assessed, and similar cuts of the joints were chosen. An *x*-*y* graph was drawn according to set anatomic points adapted from Arayasantiparb Tsuchimochi’s [10] approach, to be used for comparison of the pre and post-injection disc position. Distances between the disc points were calculated by the distance formula √(a_2_ − a_1_)^2^ + (b_2_ − b_1_)^2^ (diagrammatic explanation and MRI application presented later).

The mathematical formula was used to determine the difference between the most anterior points of the articular disc points, i.e., the coordinates of the anterior end of the disc were compared before and after injection. The assessor of the results was blinded to the status of the participants to avoid bias. This was repeated for the most posterior point of the articular disc. All records were also recorded before and after injection, tabulated, and statistically analysed. The mean and standard deviation values were calculated for each group in each test and normality within the data was assessed using Kolmogorov-Smirnov and Shapiro-Wilk tests.

MRI, maximum interincisal opening, and range of lateral movement data showed parametric (normal) distribution. While presence of click, NRS scale, and muscles pain showed non-parametric (not-normal) distribution *For parametric data,* independent sample *t*-test was used to compare between two groups in non-related samples. Repeated measure ANOVA was used to compare between more than two groups in related samples. *For non-parametric data,* Mann-Whitney test was used to compare between two non-related samples. Freidman test was used to compare between more than two groups in related samples. The significance level was set at *P* ≤ 0.05. Statistical analysis was performed with IBM® SPSS® Statistics Version 20 for Windows.

## Results

The patients included in this study were females with bilateral painful clicking joints with no limitation of movement or muscular disorders. The diagnosis of ADD with reduction was reached clinically and confirmed by MRI. Patients of both groups I and II received identical BTX-A injections and none of them showed any post-injection complications.

### Clinical results

**Fig. 4:**
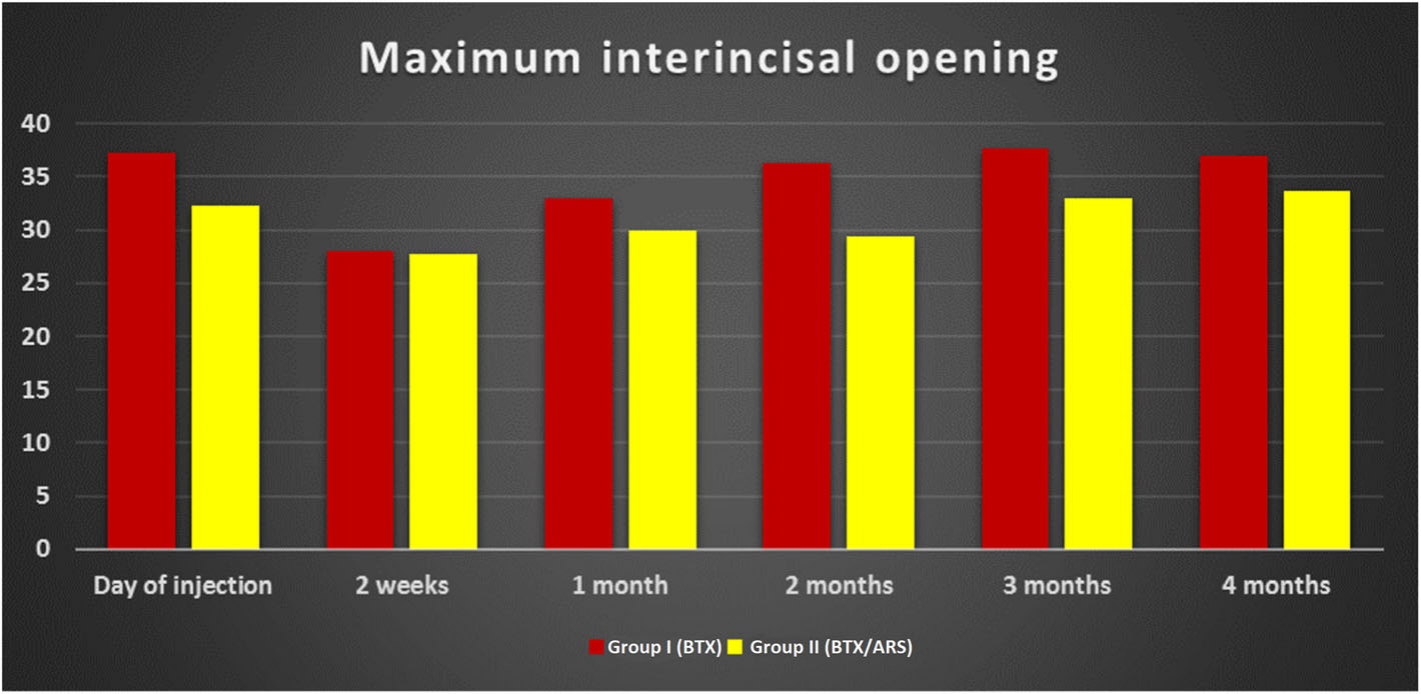
Bar graph showing the maximal interincisal opening in millimetres along the study period in both groups

Regarding the maximal interincisal opening, a statistically significant difference between the reading of groups I and II was only recorded on the 2nd month with a *p*-value of 0.011, while throughout the rest of the study period no statistical difference between the groups was noted. The interincisal opening dropped greatly 2 weeks after injection (statistically significant difference with the preoperative reading) but was regained gradually by the end of the study in both groups (Fig. 4).

**Fig. 5:**
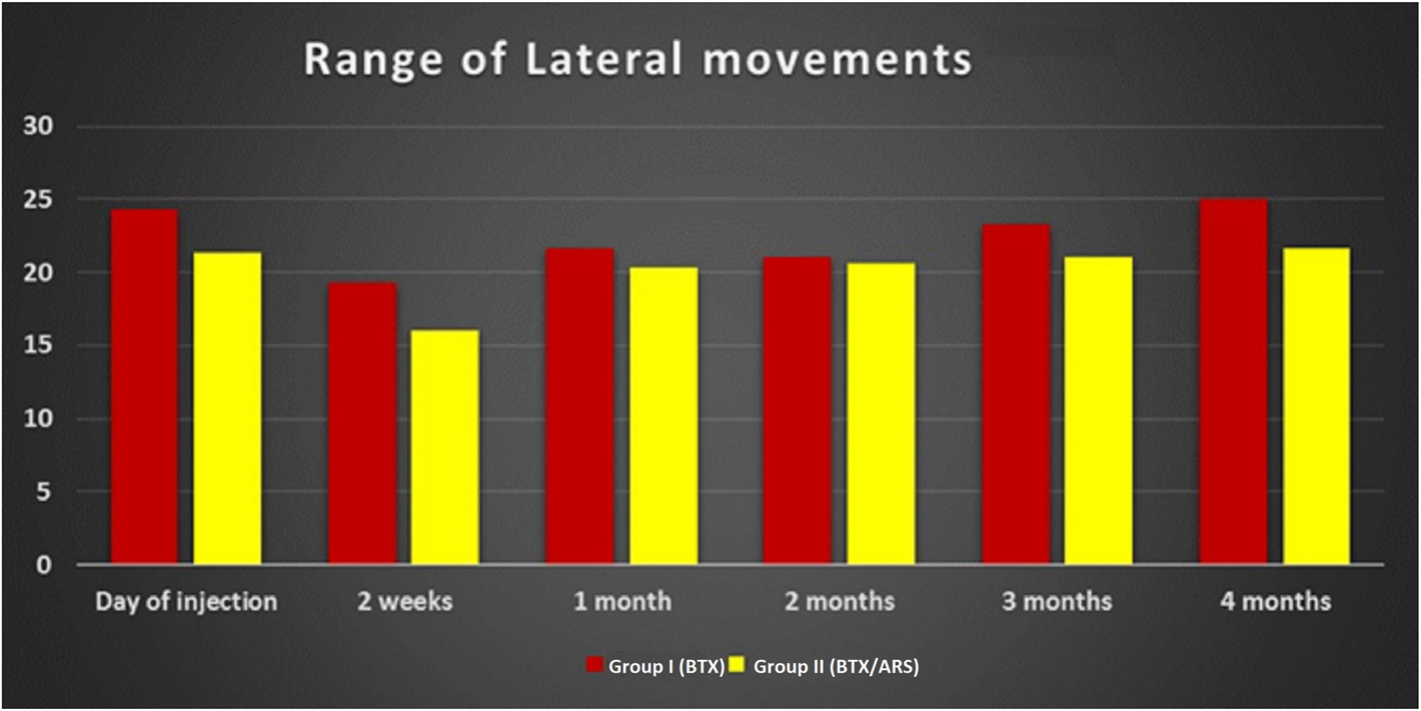
Bar graph showing the lateral movement in millimetres along the study period in both groups

The range of lateral movement showed a significant drop 2 weeks after injection and then gradually increased. The lateral range of movement increased greatly in group I than group II only at the 4 month follow-up visit with a mean of 25 mm in group I and 21.6 mm in group II. In both groups, the preoperative range of lateral movement was regained by the end of the 4-month follow-up (Fig. 5)

Regarding pain score assessment, there was no statistically significant difference in the recorded mean pain scores between the groups. Despite that, there was a big decrease in mean pain score from 5.3 and 6.7 (group I and II preoperatively) to 1.6 in both groups post-injection (Table 1).

**Table 1:** The changes and differences in the pain scores according to the visual analogue scale throughout the study period (*SD* standard deviation, *ns* non-significant)

Variables	NRS				*p-value*
	Group 1		Group 2		
	Mean	SD	Mean	SD	
**Day of injection**	5.33	1.37	7.67	1.86	**0.065 ns**
**2 weeks**	2.67	1.86	5.33	1.37	**0.065 ns**
**1 month**	2.00	1.55	3.00	1.79	**0.394 ns**
**2 months**	1.33	0.52	2.00	0.89	**0.240 ns**
**3 months**	1.33	0.52	1.67	1.03	**0.818 ns**
**4 months**	1.67	0.52	1.67	1.03	**0.818 ns**
***p-value***	**0.0**	**01**^a^	**≤ 0.**	**001**^a^	

^a^Significant

**Fig. 6:**
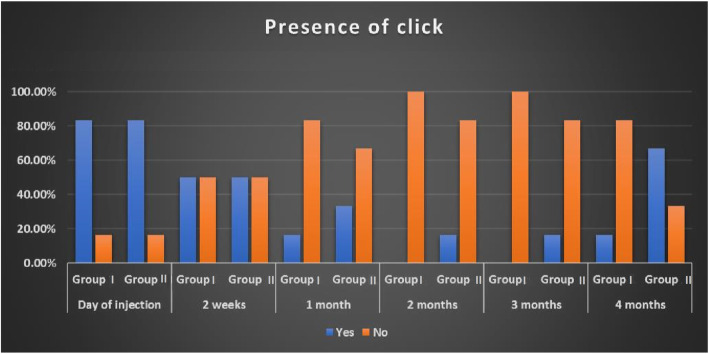
Bar graph showing the presence of a click during the study period in both groups

Regarding the presence of the TMJ click, all 12 joints had a preoperative click. Fifty percent of the joints included in both groups lost the click at the 2nd week post-injection. All clicks disappeared in group I after 3 months, while 1 joint had a click by the 4th month. In group II one joint regained the click by the 2nd month. Four months after injection four of the joints of group II regained their clicks (Fig. 6).

### MRI results

**Fig. 7:**
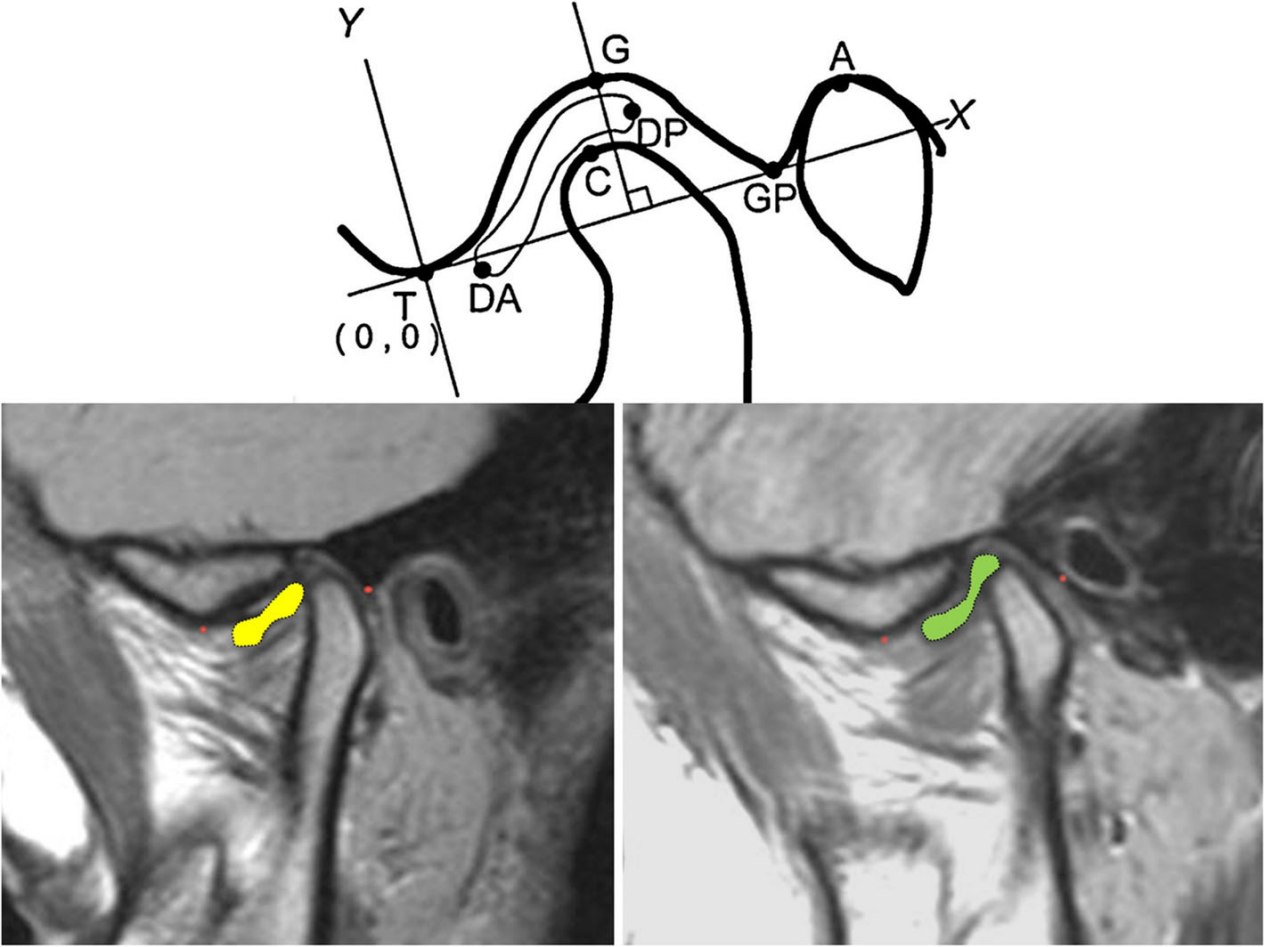
Image showing a graphical representation of the point system used to locate the articular disc on the MRI (adapted from Arayasantiparb et al. [1] [top]). MRIs of one of our patients, preoperative left and postoperative left. Red points identify the T (lowest point of eminence will mark (0,0) on the (*x*,*y*) graph) and GP (posterior glenoid tubercle) (will mark (10,0) point of the (*x*,*y*) graph to be drawn). The discs are coloured for visualization [bottom]. Point DA, anterior-most convexity of the articular disc; point DP, posterior most convexity of the articular disc; point C, uppermost point of the mandibular condyle; point GP, lower-most point of the posterior glenoid tubercle; point A, uppermost point of the external auditory meatus

To assess the change in disc position the anterior and posterior most points of the articular disc were assigned (*x*,*y*) coordinates preoperatively. The same points were given coordinates on the 4-month follow-up MRI. The distance formula was then applied to these coordinates to identify change in position of these points and the mean for each group was calculated. A statistically significant difference was found between groups I and II with a *p* value of 0.027. The most significant position change value was noted in group I (1.33 ± 0.76 mm), while the lowest value was in group II (0.64 ± 0.23 mm). Figure 6 shows the diagrammatic explanation and an example of its application on one of the joints of group I (Fig. 7).

## Discussion

Conservative management protocols, including splint therapy, for ADD are always the treatment option to start with [11–13]. Initial diagnosis of ADD with reduction in this study was achieved clinically by pre-auricular palpation of the joints. Definitive diagnosis used MRIs to locate the anteriorly displaced articular disc in the closed mouth position and disc reduction in the open mouth position. Although other reports have introduced other imaging modalities (radiographs and ultrasound), the accuracy of ADD diagnosis with MRI has been superior [14, 15] and it remains the most accurate especially in diagnosing anterior disc displacement with reduction. MR assessment is one of the main methods to visualize and assess disc status and position [14]. MRI for initial diagnosis was reported as an extreme measure by some, stating that TMD diagnosis should sufficiently be based on clinical signs, [16] while others stated imaging necessary to avoid misdiagnosis [17]. In our study, we depended on MRI to confirm clinical diagnosis and allow comparing pre- and post-injection disc position. Visual assessment of the position of the disc is usually sufficient for diagnosis, but for the sake of quantitative assessment of disc position an (*x*,*y*) graph was used as proposed by Arayasantiparb et al. [10, 18]. To allow for the comparison of disc position, a distance formula was applied as proposed by Emara et al. [8]. This formula calculates the coordinate change of the points (most anterior and posterior points) of the articular disc before injection and 4 months after it. In our study, both groups showed some backward repositioning of the articular disc; however, group I showed significant disc position improvement.

35 U of BTX-A was injected in the lower head of the lateral pterygoid muscle in the current study as reported by other research groups [5, 8]. Reports of using 50 U were accompanied with higher rates of complications such as dysphagia and botulism [19]. Since the lateral pterygoid muscle is a relatively small, thin muscle, it requires smaller BTX dosage than other muscles (such as the masseter) and a single-point injection is sufficient [5, 8]. To ensure intramuscular injection, identification of the lateral pterygoid muscle was confirmed by an audible electromyogram as recommended [5, 8, 20]. Although other technologies were reported for injection guidance — such as CT-guided, MR-guided, and arthroscopic guidance [20, 21]; EMG is the easiest and most affordable. Once in the target position, the patient was asked to perform a lateral mandibular putting the lateral pterygoid muscle in action, confirmed by the EMG’s distinct beep and so ensuring an intramuscular injection.

BTX causes temporary chemical denervation where the pre-synaptic release of neurotransmitters is blocked, preventing muscle contraction. On that basis, many craniofacial disorders such as dystonia and mandibular dislocation have been treated by BTX injection [19, 22, 23]. One of the theories for anterior disc displacement is hyperfunction of the lateral pterygoid causing the anterior pull on the disc to surpass the backward pull of the elastic retrodiscal tissues and so displacing the disc anteriorly [8]. The lateral pterygoid muscle consists of two heads; the upper head which inserts into the disc/capsule complex and the lower head which inserts into the neck of the mandible [24]. Although the injection in the current study was in the lower head, the function of the whole muscle was blocked; this was seen clinically as the lateral movements were reduced. This may support the theory that the two heads of the muscle are of the same origin and are basically a single motor unit. This is in accordance with earlier reports concluding the relation of both heads of the muscle [8].

The effect of the BTX peaks after about 2 weeks and wears off by the 4th month as stated by the manufacturer and in literature [5, 8, 22, 23]. This agrees with the results of the present study, where the maximal drop of mandibular movement (interincisal or lateral movement) was after 2 weeks. By the end of the study period, one joint in group I regained the click while four joints in group II had a click. This supports the hypothesis that BTX relieved the lateral pterygoid muscle’s anterior pull giving the disc a chance to regain its location or at least improve it [5, 8]. When the articular disc moved further posterior on the condylar head; this was considered a disc position improvement. This also relieves the stress on the retrodiscal tissues and alleviates the pain. Reports of some long-term muscular changes that may occur due to BTX injection may explain why some cases require a second injection while others lost all signs and symptoms with a single injection [22]. The patients of this study are still followed up to identify long-term results.

In the current study, group II patients received an anterior repositioning splint to assess the effect that would have on the already proven effect of BTX. This however was not proven since 4 joints regained clicks by the end of the follow-up period and the patients reported a higher pain scores than group I. This may be explained by the discomfort the patients reported from the occlusal devices which led to stress and so a lower pain threshold. The patients were annoyed that they had to wear a splint during sleep and it caused more salivation that lead to sleep disturbances. Improper sleep schedule may have resulted in an inferior psychological state. The use of anterior repositioning splints was proposed by Jeffrey Okeson [25] and successfully reported by others [25, 26] aiming to allow disc recapture. Weinberg and Lager [27] suggested positioning the condyle in a more therapeutic anterior position to allow re-capturing of the anteriorly displaced disc. This was achieved by an acrylic ramp fabricated to guide the mandible to a favourable condylar position. Our results, which negate the importance of these splints, align with other researchers revoking their use for the management of internal derangement [28, 29].

Pain scores were assessed using the NRS as was reported several times and aim to assess quality of life [30–32]. Pain scores in our study were consistent with other results, where the maximum drop in score was simultaneous to the peak of BTX action, 2 weeks post-injection. The pain scores slowly increased, but by the end of the follow-up period remained lower than those recorded preoperatively. Group II patients had higher pain scores than group I patients; this may be attributed to the lower psychological state these patients reported due to discomfort from the splint.

All participants reported improved joint condition by the end of the study period. Group II patients generally had more complaints and discomfort. The clicks disappeared in both groups after the second month of injection. This supports the hypothesis that the lateral pterygoid muscle has an anterior pull on the disc and may be a cause of disc displacement when it is in hyperfunction. When the action of the lateral pterygoid was negated (by the BTX injection) the disc was able to regain a better position.

## Conclusions

Although the results are slightly in favour of injecting BTX alone, as viewed by magnetic resonance imaging, however BTX injection is an expensive invasive approach that needs EMG guidance to accurately reach the lateral pterygoid muscle. Reports on BTX injection being a temporary muscle relaxant that fades in a period of 4 to 6 months and patients may regain clicks may also discourage against using it. On the other hand, splint fabrication is less expensive and complex although it causes discomfort and patient distress. The clinician must therefore weigh the patient’s needs and cost-effectiveness. With this study being centred in an Egyptian public university hospital; cost of the treatment provided is a major issue.

Further studies into the exact action of the lateral pterygoid muscle and the use of BTX in different forms of TMDs are necessary required. Larger sample sizes with longer follow-up periods are needed to study the effect of BTX and the long-term effects on the muscles and surrounding structures. Introducing new modifications in the design of anterior repositioning splints should also be attempted to reduce the discomfort it causes.

## Data Availability

Available

## Abbreviations

ADD: Anterior disc displacement
BTX: Botulinum toxin
CT: Computed tomography
EMG: Electromyography
MRI: Magnetic resonance imaging
NRS: Numerical rating scale
TMDs: Temporomandibular disorders

## Footnotes

1 Signal Amplifier for BTX-A, manufactured by Barrett Engineering Fortuna, CA 95540. US distributor Allergan, Inc., Irvine, CA 92612, USA
